# Traffic flow prediction using bi-directional gated recurrent unit method

**DOI:** 10.1007/s44212-022-00015-z

**Published:** 2022-12-01

**Authors:** Shengyou Wang, Chunfu Shao, Jie Zhang, Yan Zheng, Meng Meng

**Affiliations:** 1 School of Traffic Management, People’s Public Security University of China, Beijing, 10038 China; 2 Key Laboratory of Transport Industry of Big Data Application Technologies for Comprehensive Transport, Beijing Jiaotong University, Beijing, 10044 China; 3 Business School, University of Bristol, Bristol, BS8 1SD UK; 4 School of Transportation, Southeast University, Nanjing, 211189 China; 5 School of Management, University of Bath, Bath, BA2 7AY UK

**Keywords:** Short-term traffic flow prediction, Deep learning method, Urban expressway, Bi-GRU

## Abstract

Traffic flow prediction plays an important role in intelligent transportation systems. To accurately capture the complex non-linear temporal characteristics of traffic flow, this paper adopts a Bi-directional Gated Recurrent Unit (Bi-GRU) model in traffic flow prediction. Compared to Gated Recurrent Unit (GRU), which can memorize information from the previous sequence, this model can memorize the traffic flow information in both previous and subsequent sequence. To demonstrate the model’s performance, a set of real case data at 1-hour intervals from 5 working days was used, wherein the dataset was separated into training and validation. To improve data quality, an augmented dickey-fuller unit root test and differential processing were performed before model training. Four benchmark models were used, including the Autoregressive Integrated Moving Average (ARIMA), Long Short-Term Memory (LSTM), Bidirectional Long Short-Term Memory (Bi-LSTM), and GRU. The prediction results show the superior performance of Bi-GRU. The Root Mean Square Error (RMSE), Mean Absolute Percentage Error (MAPE), and Mean Absolute Error (MAE) of the Bi-GRU model are 30.38, 9.88%, and 23.35, respectively. The prediction accuracy of LSTM, Bi-LSTM, GRU, and Bi-GRU, which belong to deep learning methods, is significantly higher than that of the traditional ARIMA model. The MAPE difference of Bi-GRU and GRU is 0.48% which is a small prediction error value. The results show that the prediction accuracy of the peak period is higher than that of the low peak. The Bi-GRU model has a certain lag on traffic flow prediction.

## Introduction

Accurate short-term traffic flow prediction is crucial for transportation management. The main task of short-term traffic flow prediction is to forecast the next step of traffic flow based on historical traffic data (Nagy & Simon, 2018; Wu et al, 2018). The results from traffic flow prediction are served as a reference for both travel demand analysis and operation strategy development. Moreover, traffic flow prediction model is also one of the key components for smart transportation systems. Having the accurate predicted results is the fundamental basis to decide how to guide the optimal travel routes for traveler so that to reduce the traffic congestion and improve the traffic efficiency and safety.

Some classical traffic flow prediction models including Historical Average (HA) model, Autoregressive Integrated Moving Average (ARIMA) model (Van Der Voort et al, 1996), and Linear regression (LR) (Haoyi & Jing, 2011) have been well applied in practice. These models are simple in structure but have the disadvantage that the fluctuations in traffic flow can significantly affect the prediction performance. Some scholars have applied Support Vector Regression (SVR) model (Cheng et al, 2017; Wei & Liu, 2013) and Back Propagation Neural Network (BPNN) model (Kumar & Katiyar, 2013) in traffic flow prediction. These models have good applicability to complex situation, but also have certain drawbacks like complex model structure, large computational effort, and difficulty in determining model parameters.

In recent years, the rapid development of data collection and computing technologies has greatly improved the performance of short-term traffic flow prediction (Chen, Liu, et al., 2021; Wang et al, 2022). Deep learning method, which is one of the current leading techniques for short-term traffic flow prediction, has been widely proposed and applied in ITS. Deep learning method can be divided into tress branches. One branch is designed for mining spatial characteristics, such as Convolution Neural Network (CNN) (Cao & Wang, 2019), Graph Convolutional Networks (GCN) (Wang et al, 2022). One is proposed for extracting the temporal characteristics, such as Recurrent Neural Network (RNN) (Duives et al, 2019), Long Short Term Memory (LSTM) neural network, Bidirectional Long Term Memory (Bi-LSTM) neural network (Ma et al, 2022), Gate Recurrent Unit (GRU) neural network (Shu et al, 2022); Others have the generative adversarial network (GAN) (Zhu et al, 2020), AutoEncoder (Wei et al, 2019). Since short-term traffic flow prediction is estimated by learning the temporal characteristics of historical observation, which belongs to the time series prediction problem, we mainly focus on time series-related deep learning methods.

Among the methods above, RNN is proposed firstly for the extraction non-linear features of time sequences, and has the advantage of strong memory, and sharing parameters, which have been proven to greatly improve the accuracy of short-term traffic flow prediction compared with the traditional parameters models, such as HA, ARIMA and the Kalman Filter (KF) model (Gu et al, 2019; Tedjopurnomo et al, 2022). However, RNN has the disadvantage of gradient disappearance and gradient explosion, which cannot learn the long-term dependencies of the time-series data well. Therefore, to address the problem of RNN, the variant of RNN, LSTM is proposed by adding three gates (e.g. input gate, out gate, forget gate) in the hidden layer of RNN to control the retention and forgetting of information, and further memory the long-term (e.g. 12 hour, 1 day) and short-term (e.g. 1 hour, 2 hours) information of time series (Bogaerts et al, 2020; Kim & Lee, 2022; Yang, Chen, et al., 2019). Therefore, LSTM can memory more comprehensive time characteristics than RNN. LSTM has been applied to short-term traffic flow prediction, and has shown more effective prediction performance than RNN (Yang, Sun, et al., 2019). Subsequently, the Bi-LSTM is proposed combined two LSTM layers in opposite directions for mining the sequential and inverse-order time series information, which has been applied in traffic flow prediction (Ma et al, 2022). However, since the LSTM and Bi-LSTM have three gates in the hidden layer, which requires a large number of parameters and time for training and fitting, scholars streamlined the complex structure of the LSTM model and proposed GRU with two gates of hidden layer to improve the model efficiency (Shahid et al, 2020). Researchers applied the GRU on traffic flow prediction and showed a higher prediction efficient than LSTM, and a higher accuracy rate than RNN (Sun & Tao, 2020). Similar to the Bi-LSTM, Bidirectional Gated Recurrent Unit (Bi-GRU) neural network (Huang et al, 2021), which consists of two GRU layers with opposite directions, was subsequently proposed and proved to be effective in the natural language domain (Li et al, 2020). However, few literatures have applied the Bi-GRU in short-term traffic flow prediction to demonstrate its prediction performance.

As reviewed, Bi-GRU, LSTM, Bi-LSTM, and GRU belong to recurrent neural networks which play a key role in the field of time series prediction. When designing a combined model of traffic flow prediction, Bi-GRU, LSTM, Bi-LSTM, and GRU could be selected as the parts for extracting temporal characteristics. However, do these models have some similar prediction results? What are the differences in the prediction performance of these models for the traffic flow prediction problem? Is there a model among these models recommended for urban managers in terms of traffic flow prediction? To answer these questions, we calibrate and validate the Bi-GRU model using real traffic flow data, and test the prediction performance in two scenarios. In summary, the contribution of this paper is twofold: on the one hand, we apply the Bi-GRU model in short-term traffic flow prediction and discuss its prediction performance compared with LSTM, Bi-LSTM, and GRU models. On the other hand, we further explored the performance of Bi-GRU for short-term traffic flow prediction model in peak and low-peak periods on each road sections, which can demonstrate the results from both spatial and temporal aspects. The research outcome will provide references for researchers and managers in selecting traffic flow prediction models associated with recurrent neural networks.

The rest of the paper is organized as follows: Section 2 summarizes the existing literature. Section 3 explains the proposed model in detail. And in section 4, we describe the experimental data and model evaluation methods. In Section 5, the predictive performance of the proposed model is evaluated and the model output is analyzed. Finally, Section 6 presents the main conclusions and future directions of this study.

## Literature review

### Short-term traffic flow prediction

As a hot research topic in the transportation system, short-term traffic flow prediction methods have achieved rich research results in the past 50 years (Nagy & Simon, 2018). The methods of short-term traffic flow prediction can be broadly classified into traditional parametric, and non-parametric models (Kaffash et al, 2021).

The traditional parametric models have been widely applied to address short-term traffic flow prediction problems (Wu et al, 2014). In general, parametric models assume that the time-varying traffic flows obey one or several distributions and predict traffic flow by parameter fitting. Among parametric models, the ARIMA model (Williams, 2001), LR and KF model (Xie et al, 2007) have satisfactory applicability to traffic flow prediction problems. For example, Yao et al (2016) combined the K-nearest neighbors (KNN) method and the KF technique to dynamically predict real-time traffic flow. In the numerical test, the proposed model performed better than a single KNN model. Li (2020) applied the multiple linear regression model for short-term traffic flow prediction in urban. The experimental results showed that, compared with decision tree methods (Kamiński et al, 2018), The proposed model has a higher prediction accuracy of 98.48% and a shorter prediction time, always less than 0.7 seconds. In addition, some scholars have combined multiple parametric models to improve traffic flow prediction accuracy. For example, Xu et al (2017) combined KF with ARIMA to achieve traffic flow state prediction of road sections.

Although parametric models have better prediction accuracy compared with statistical models, they still cannot fully adapt to the strong randomness of traffic flow. With the rapid development of computer technology, non-parametric models have gradually occupied the dominant position in the field of short-term traffic flow prediction. Models such as K-Nearest Neighbor (KNN) model (Luo et al, 2019; Zhang et al, 2013), SVR model (Zhang et al, 2018), BPNN model, Fuzzy Neural Networks (FNN) models (Moretti et al, 2015) have been proven to give promising results in traffic flow prediction problems. For example, Sun et al (2018) proposed a fully automatic dynamic procedure KNN to predict traffic flow. The results show that the proposed model performed better than the normal KNN and seasonal ARIMA (Shu, 2005) in terms of accuracy on average. Li and Xu (2021) applied SVR for the short-term traffic flow prediction. The results were obtained from experiments that the prediction error rate was the lowest (3.22%) compared with RF and Adboost (Kanduri et al, 2018). Zhang and Qu (2021) proposed a GA-BPNN model combining an adaptive genetic algorithm (Li et al, 2004) and BPNN to predict short-term traffic flow. The results show that the average prediction error of the proposed algorithm is about 1%, and the computational accuracy is better compared with that of a single BPNN.

### Recurrent neural network variants

As mentioned, short-term traffic flow prediction belongs to a time series prediction problem and the deep learning method of RNN is designed to deal with this problem and has a wide range of applications (Zhang et al, 2014). For example, Chen et al (2020) proposed an attention-based RNN model for multi-step traffic flow prediction. Experimental results demonstrated that the proposed model had good performance compared to the KNN, and sequence to sequence (seq2seq) (Zhang et al, 2019). However, RNN has several disadvantages such as gradient disappearance, gradient explosion. To deal with these problems of RNN, scholars proposed many variants based on RNN, where LSTM, Bi-LSTM, GRU, and Bi-GRU have a wide application (Tedjopurnomo et al, 2022; Zhang et al, 2021).

LSTM modifies the hidden layer of RNN to gain the advantage of long-term memory, and had been introduced to short-term traffic prediction. Yang, Chen, et al. (2019) introduced the LSTM model for short-term traffic flow prediction, and the results showed that the proposed model had certain competitiveness in short-term traffic flow predictions. Xiao and Yin (2019) proposed a hybrid LSTM neural network to predict traffic flow. The results found that the prediction error of the proposed model was less than KF and SVR. For short-term traffic flow prediction, Zheng et al (2021) proposed a deep learning based model combined the convolutional neural network (CNN) and the LSTM to extract the spatial and short-term temporal features. Extensive experimental results showed that the proposed model achieved better prediction performance compared with SVR.

Similar to LSTM, Bi-LSTM extracts the temporal characteristics by two LSTM layers in opposite directions, which have been applied in short-term traffic flow prediction. Abduljabbar et al (2021) introduced the Bi-LSTM model for short-term traffic flow prediction. The results showed that the Bi-LSTM performed better than LSTM. Li et al (2021) introduced Bi-LSTM for traffic flow prediction, and applied GRU and LR in the experiment as a comparison. The experimental findings demonstrated that the Bi-LSTM model worked best in predicting traffic flow, achieving an accuracy of 92% when temporal differences were taken into account. Xing and Liu (2022) constructed a data fusion powered Bi-LSTM model for traffic flow prediction. The results showed that the proposed model produced more accurate predictions compared with LSTM, Bi-LSTM, GRU. However, he did not evaluate the prediction performance of Bi-GRU.

GRU is another variant of RNN with fewer parameters than LSTM, which has been introduced for short-term traffic flow prediction. For example, Zhang and Kabuka (2018) applied GRU model for short-term traffic flow prediction. The results showed that the GRU performed better than ARIMA, SVR, and RF. However, he did not evaluate the performance of GRU in comparison with LSTM, and Bi-LSTM. Wang et al (2020) applied GRU and LSTM at the same time for truck traffic flow prediction. The results showed that LSTM and GRU have superior performance compared to SVR and ARIMA. In addition, The overall accuracy of LSTM was 4.10% higher than that of GRU. Dai et al (2019) applied GRU for short-term traffic flow prediction. The results showed that the proposed method outperformed CNN in terms of accuracy and stability. However, he did not test the performance of LSTM.

Compared to LSTM, Bi-LSTM, and GRU, Bi-GRU is the latest to be proposed which combined with two GRU layers in opposite directions. Bi-GRU has been introduced in many prediction problems (e.g. wind power prediction, COVID-19 cases prediction, oil rate prediction). For example, Chen, Qi, et al. (2021) applied Bi-GRU to predict wind power, and the results proved its superior prediction performance compared with LSTM and GRU. Ahuja et al (2022) used CNN and stacked Bi-GRU to predict the COVID-19 cases. The experimental result showed that the proposed model was highly reliable over the gaussian process regression model (Schulz et al., 2018). Li et al (2022) proposed a framework using Bi-GRU and sparrow search algorithm (Zhang et al, 2022) to improve the accuracy of oil rate prediction. The observations showed that the proposed method performed better than RNN, LSTM, and GRU in terms of accuracy and robustness. Other than that, Shu et al (2022) have introduced the Bi-GRU for short-term traffic flow prediction and showed that the Bi-GRU performed better than LSTM. However, he did not discuss the model performance in comparison with GRU, and Bi-LSTM. Further, few scholars pay attention to the prediction performance of Bi-GRU during peak and low-peak periods of traffic flow.

In summary, traffic flow has complex temporal relationships, and scholars mainly use the data of time series information for prediction. Bi-GRU model has been proven the good performance in short-term traffic flow prediction, but few scholars evaluate the Bi-GRU prediction performance in comparison with LSTM, Bi-LSTM, GRU under the same dataset. Therefore, this paper introduce the Bi-GRU model to capture the temporal characteristics for traffic flow prediction, and discuss its prediction performance compared with LSTM, Bi-LSTM, and GRU models. Furthermore, we explored the performance of Bi-GRU for short-term traffic flow prediction model in peak and low-peak periods on each road sections, which can demonstrate the results from both spatial and temporal aspects. The discussion in this paper will provide some references for researchers and managers in selecting traffic flow prediction models associated with recurrent neural networks. In addition, accurate traffic flow forecasts will provide useful information for urban managers to take control measures and for residents to plan their travel routes.

## Methodology

### Bi-directional gated recurrent unit (bi-GRU) model

Bi-GRU model is a variant of RNN, which have capacities to memory long-term dependencies (e.g. 1 day traffic flow information at 1 hour interval) of time series data (Wang, Shao, et al., 2021). Short-term traffic flow prediction belongs to time series prediction problem, which indicates that Bi-GRU can be applied to short-term traffic flow prediction. Bi-GRU is composed of forward GRU and backward GRU. Compared with LSTM, GRU has a less complex structure and higher computational efficiency (Greff et al, 2017). The structures of the GRU is described by Cho et al (2014), which includes the input layer, the hidden layer, and the output layer. The hidden layer is composed of the reset gate and the update gate, which is used to control the information of traffic flow from the input layer at time *t* and the hidden layer at time *t* ‐ 1 (Agarap, 2018). We define the traffic flow input data of a road section as *x*_*t*_. *t* = (1, 2, …, *n*), is the number of observed traffic flow records during the period indexed in time order, where the period implies the time length of the traffic flow data recorded. The output of GRU is defined as *h*_*t*_, the output of reset is defined as *r*_*t*_, and the output of update gate is defined as *z*_*t*_. Moreover, the reset and update gates calculate the output *h*_*t*_ of the current moment by the joint control of the output *h*_*t* − 1_ of the previous moment and the input *x*_*t*_ of the current moment. The equations of reset gate and update gate are shown in Eq. (1) and Eq. (2).

1 $${r}_t=\sigma \left({W}_r\cdot \left[{h}_{t-1},{x}_t\right]\right)$$

2 $${z}_t=\sigma \left({W}_z\cdot \left[{h}_{t-1},{x}_t\right]\right)$$

where, *W*_*r*_ and *W*_*z*_ are the weights of the reset gate and the update gate respectively, and *σ* is the Sigmoid function, where, *σ*(*x*) = 1/(1 + *e*^−*x*^). The calculation equation of output *h*_*t*_ is shown in Eq. (3).

3 $${h}_t=\left(1-{z}_t\right)\times {h}_{t-1}+{z}_t\times {\tilde{h}}_t$$

where, ${\tilde{h}}_t$ is the candidate state of GRU at time *t*. The calculation of ${\tilde{h}}_t$ is shown in Eq. (4).

4 $${\tilde{h}}_t=\tanh \left({W}_h\cdot \left[{r}_t\times {h}_{t-1},{x}_t\right]\right)$$

where, *W*_*h*_ is the weight of the candidate state.

As mentioned, the Bi-GRU is constructed by two unidirectional GRUs facing opposing directions (Xiong et al, 2016). The forward GRU starts from the beginning of the time series data, and the backward GRU starts form the end of the time series data. The Bi-GRU is calculated by two GRU can be formulated as Eq. (5)–(7).

5 $${\overrightarrow{h}}_t={GRU}_{fwd}\left({x}_t,{\overrightarrow{h}}_{t-1}\right)$$

6 $${\overleftarrow{h}}_t={GRU}_{bwd}\left({x}_t,{\overleftarrow{h}}_{t-1}\right)$$

7 $${h}_t={\overrightarrow{h}}_t\oplus {\overleftarrow{h}}_t$$

where, ${\overrightarrow{h}}_t$ and ${\overleftarrow{h}}_t$ are the state information of the forward and backward GRU, respectively. *GRU*_*fwd*_ is the forward GRU, and *GRU*_*bwd*_ is the backward GRU, the GRU function is composed of Eq. (1) - Eq. (4). ⊕ denotes concatenating the ${\overrightarrow{h}}_t$ and ${\overleftarrow{h}}_t$. Therefore, the Bi-GRU with bi-directional GRU structures can memory the traffic flow information from historical and subsequent time series data.

### Comparison models

**Fig. 1:**
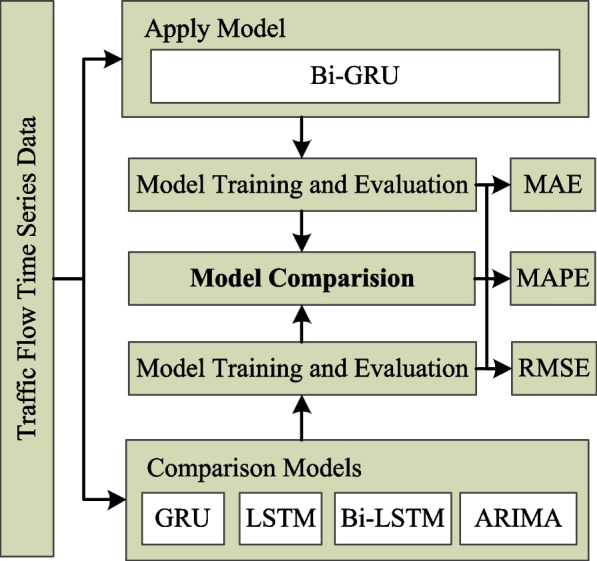
Flow chart of the paper structure

In this paper, four benchmark methods including ARIMA, LSTM, GRU, and Bi-LSTM are selected for comparison, which have the ability to mine the temporal characteristics of time series data, and have been applied to short-term traffic flow prediction in existing literature (Shuai et al, 2022; Zhao et al, 2021). The process of the model comparison part among this paper can be seen in Fig. 1.

The details of the benchmark methods are described as follows:

1. Autoregressive Integrated Moving Average (ARIMA) is the representative model of the traditional parameters model, which can mine temporal features by statistical approaches for traffic flow prediction (Meng et al, 2015; Xu et al, 2017). Compared with Bi-GRU, we can explore whether the prediction performance of Bi-GRU is better than that of traditional models.
2. Long Short-Term Memory (LSTM) is the classical variant model based on RNN (Yang, Sun, et al., 2019), which is widely used and outperform in short-term traffic flow prediction. Compared with Bi-GRU, it only considers the influence of past information on the prediction time. Meanwhile, LSTM is more complex than GRU in structure.
3. Gated Recurrent Unit (GRU) is the classical variant model based on RNN (Zhang & Kabuka, 2018). Compared with LSTM, it has fewer parameters and compared with Bi-GRU, it only consider the influence of past information on the prediction time.
4. Bidirectional Long Short-Term Memory (Bi-LSTM) contains three gating units, which are input gate, output gate and forget gate (Ali et al, 2021). The input gate controls the input transmission of traffic flow, the forget gate controls whether the information of the memory module is transmitted, and the output gate is used to determine the output of the information. Bi-LSTM has been applied to short-term traffic flow prediction, and it has been proven to achieve a high prediction accuracy. Compared with Bi-LSTM, Bi-GRU has a simpler structure and has been proved to have a considerable prediction performance in natural language applications.

It should be noted that models (2)–(4) have the same basic parameters settings: the number of hidden neurons is set to 64, the batch size is set to 10, the number of iterations is set to 200, the learning rate is set to 0.01, and the time step is set to 9. In addition, these models use the Adam optimizer to minimize the loss function during training, and the early stopping is set to to prevent overfitting. Moreover, the deep learning packages of Tensorflow and Keras (Pang et al, 2020) are used. A general-purpose programming language is provided by Python 3.6. In addition, for ARIMA, the auto_arima is applied to automatically determine the most suitable parameters. The ‘auto_arima’ is the python package of ARIMA model, which can automatic find the optimal parameters for each road section by multiple calculating the error between predicted and observed traffic flow.

### Evaluation metrics

An excellent prediction model needs to have the ability to accurately capture the temporal characteristics from the historical traffic flow information. In this research, we apply three evaluation metrics, including the Mean Absolute Error (MAE), the Mean Absolute Percentage Error (MAPE) and the Root Mean Square Error (RMSE) to evaluate the prediction performance of the proposed short-term traffic flow prediction model (Kumar & Katiyar, 2013; Xue & Xue, 2018; Meng, Chang, et al., 2022). The calculation of MAE, MAPE and RMSE are shown in Eq. (8)-Eq. (10).

8 $$RMSE=\sqrt{\frac{1}{n}\sum \limits_{i=1}^n{\left({\hat{y}}_i-{y}_i\right)}^2}$$

9 $$MAPE=\frac{100\%}{n}\sum \limits_{i=1}^n\left|\frac{{\hat{y}}_i-{y}_i}{y_i}\right|$$

10 $$MAE=\frac{1}{n}\sum \limits_{i=1}^n\left|{\hat{y}}_i-{y}_i\right|$$

where, *y*_*i*_ represents the observation, ${\hat{y}}_i$ represents the predicted traffic flow, and *n* represents the number of traffic flow samples. Evaluation metrics are used by quantify the error between the prediction and observation. Therefore, The smaller the value of evaluation metrics the better prediction performance of the model.

## Data

### Statistical analysis

**Fig. 2:**
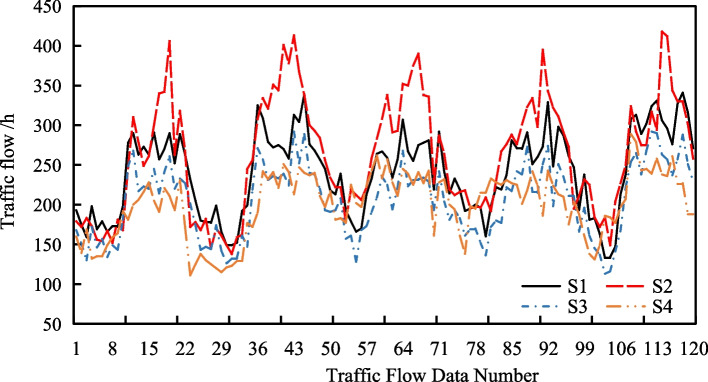
Traffic flow distribution of studied road sections

To verify the superiority of the short-term traffic flow prediction model, we use the traffic flow data for four sections of an urban expressway in Zhengzhou, China, for 5 working days from 2nd to 6th, December 2019. The time interval is 1 hour, and each section has 120 pieces of data (5 day*24 h). Particularly, the model uses the data of the first 3 days for model training, and the data of the last 2 days for model evaluation. The four road sections are numbered S1, S2, S3 and S4 respectively. The traffic flow distribution of the four road sections in 5 days is shown in Fig. 2, and the descriptive statistics of the four road sections are shown in Table 1. It can be seen from Fig. 2 that the four road sections have time periodicity, and Table 1 shows that S2 section has the highest mean and volatility of traffic flow, and S1 section has the lowest mean and volatility of traffic flow.

**Table 1:** Basic information statistics of traffic flow

Road section	Min	Max	Mean	S.D.
S1	133	341	240.61	56.36
S2	138	418	261.95	75.67
S3	113	292	204.98	49.03
S4	111	289	200.39	44.30

### Augmented dickey-fuller (ADF) unit root-based stability analysis

As can be seen in Fig. 2 and Table 1, the traffic flow data are unstable, with relatively large value differences between peak and low peak periods. Therefore, before model training, we performed ADF unit root-based stability analysis and stability processing on the traffic flow data (Aylar et al, 2019). The ADF values of the four road sections are shown in Table 2.

**Table 2:** ADF stability parameters

Index		S1	S2	S3	S4
Difference		2nd difference	1st difference	2nd difference	1st difference
Test value		−3.532	−2.512	− 3.532	− 3.532
*P*-value		0.003	0.011	0.004	0.001
Standard *P*-value	1%	−3.542	− 3.089	−2.913	− 2.531
	5%	−2.810	−2.819	− 2.438	−2.140
	10%	−2.465	−2.150	−1.883	−1.599

As shown in Table 2, after the logarithmic and differential processing of four road sections, the *p*-values of ADF are less than 0.05, which indicates that the four road sections meet the stability requirements (Zhang, 2016).

## Results and discussion

### Model performance for overall

**Fig. 3:**
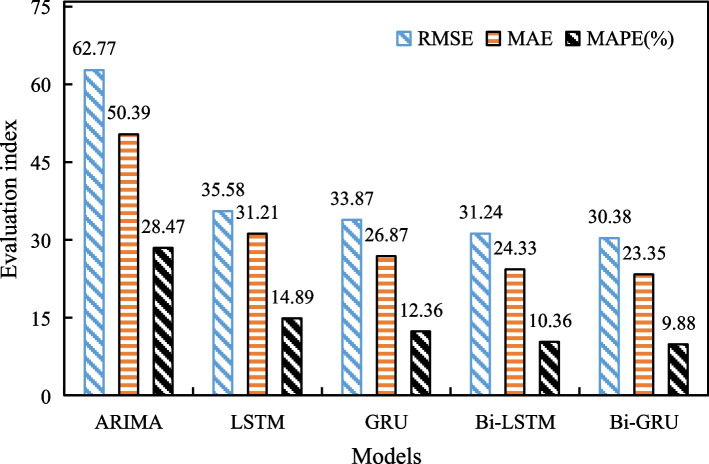
Model evaluation results

The Bi-GRU model for short-term traffic flow prediction is trained and validated, the overall evaluation results of RMSE, MAE, and MAPE are the average of four road sections, shown in Fig. 3. Meanwhile, the prediction performance of ARIMA, LSTM, Bi-LSTM, and GRU are also shown in Fig. 3 as the comparison.

It can be seen from Fig. 3 that the RMSE, MAPE and MAE values of Bi-GRU model for the overall traffic flow prediction of the four road sections are 30.38, 9.88% and 23.35, respectively, which are 0.87, 0.47% and 0.99 lower than the RMSE, MAPE and MAE of Bi-LSTM. It indicates that the overall prediction performance of the Bi-GRU model is slightly better than the Bi-LSTM model. Moreover, the prediction performance of LSTM and GRU are comparable, but both are worse than Bi-GRU and Bi-LSTM. ARIMA has the worst prediction results, and its MAE, RMSE and MAPE are significantly higher than LSTM, Bi-LSTM, GRU, Bi-GRU. This indicates that the four variants of RNN (e.g. LSTM, Bi-LSTM, GRU, Bi-GRU) which belongs to deep learning methods are is better at mining temporal characteristics than the traditional ARIMA model.

In addition, as can be seen from Fig. 3, the traffic flow prediction errors of both Bi-LSTM and Bi-GRU are smaller than those of LSTM and GRU. This indicates that the bi-directional structure of mining historical and subsequent time series data is useful for traffic flow prediction. In addition, we find that the MAPE of GRU is 12.36%, which is 2.53% lower than that of LSTM. This indicates that the GRU has a higher prediction accuracy and efficiency than LSTM for short-term traffic flow prediction. The MAPE of Bi-GRU (9.88%) is 0.48% lower than that of GRU (10.36%), which is a small difference in terms of traffic flow prediction error.

Therefore, in different application scenarios, different models can be applied for traffic flow prediction. For example, Bi-GRU is recommended for prediction in scenarios with high prediction accuracy and limited road sections. GRU is recommended for predicting traffic flow in a large road networks that require a large number of computational scenarios, combining prediction accuracy and computational efficiency.

### Model performance for road sections

The predicted evaluation results for the four road sections are shown in Table 3. Among the prediction results of the Bi-GRU model for the four road sections, the RMSE evaluation values are 32.52, 37.56, 27.92, and 23.50, respectively, which are both lower than the benchmark methods. Similarly, the MAE evaluation function values were 25.19, 27.46, 23.19, 17.55, respectively, lower than the rest of the benchmark methods. We can conclude that under the RMSE and MAE indicators, Bi-GRU model for the four road sections shows a good predictive performance.

**Table 3:** Prediction results for 4 road sections

Model	Metrics	S1	S2	S3	S4
ARIMA	RMSE	54.37	82.06	47.67	66.99
	MAPE (%)	21.93	37.09	22.71	32.16
	MAE	46.33	67.28	40.72	47.21
LSTM	RMSE	37.54	40.25	34.56	29.97
	MAPE (%)	15.87	15.22	14.51	13.96
	MAE	30.24	33.12	32.25	29.23
GRU	RMSE	33.89	37.99	32.17	31.43
	MAPE (%)	12.23	14.24	12.12	10.85
	MAE	26.45	30.58	25.97	24.48
Bi-LSTM	RMSE	33.12	37.67	28.48	25.7
	MAPE (%)	10.75	10.22	11.37	9.09
	MAE	26.62	27.78	23.89	19.04
Bi-GRU	RMSE	32.52	37.56	27.92	23.5
	MAPE (%)	9.16	11.01	10.31	9.05
	MAE	25.19	27.46	23.19	17.55

In addition, in the prediction results of the Bi-GRU model for the four road sections, except that the MAPE value of the S2 section is slightly higher than that of the Bi-LSTM, the MAPE values of the S1, S3 and S4 sections are lower than those of the other baseline models, indicating that the Bi-GRU model shows good prediction performance for most road sections. For the S2 section, the MAPE of the Bi-GRU model is 11.01% slightly higher than 10.22% of the Bi-LSTM, which may be related to the observation of the road section. The mean value and standard deviation value of S2 are 255.84 and 82.89 respectively (see Table 1), slightly higher than S1, S3 and S4. Therefore, the prediction performance of Bi-LSTM may be better than that of Bi-GRU in road sections with large flow and fluctuation.

### Model performance for peak and low peak periods

**Fig. 4:**
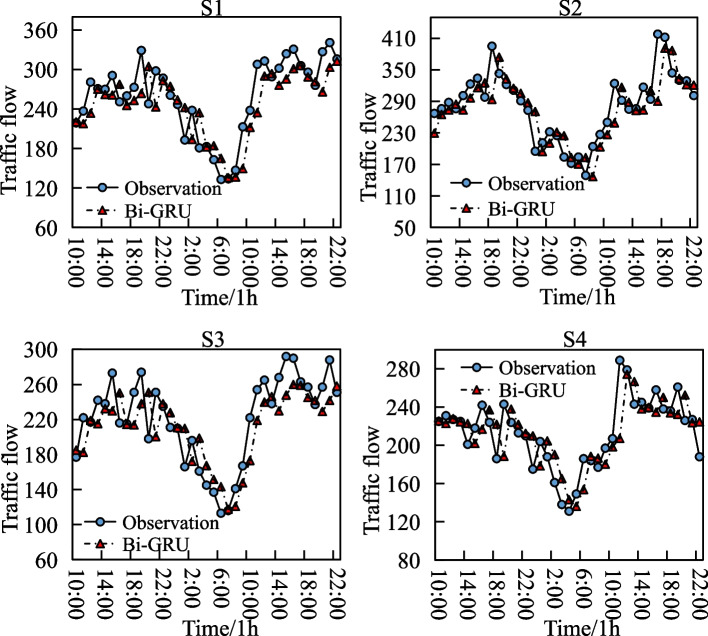
Comparison of prediction and observation for each road section

We further perform statistics on the model performance during peak (17:00–19:00) and low peak (6:00–8:00) periods (see Table 4) and compared the prediction of Bi-GRU and observation on four road sections. As can be seen from Table 4, the Bi-GRU model has lower RMSE, MAPE, and MAE in the peak period than in the low peak period, which indicates that Bi-GRU performs better in predicting traffic flow in the peak period. From Fig. 4, it can be found that in both four road sections, there has a lag in the prediction compared with the traffic flow observation. The phenomenon of lag is exhibited in each road section, which indicates the universal lag characteristics of the Bi-GRU for traffic flow prediction. This observation results is line with the findings in other articles (Yin et al, 2022).

**Table 4:** Bi-GRU performance during peak and low peak periods

Periods	Metrics	S1	S2	S3	S4	Average
Peak	RMSE	29.87	34.09	26.89	23.01	28.47
	MAPE (%)	8.93	10.89	9.91	8.95	9.67
	MAE	23.09	25.90	22.19	17.14	22.08
Low Peak	RMSE	33.91	38.35	28.43	23.78	31.12
	MAPE (%)	9.54	11.62	10.45	9.13	10.19
	MAE	26.91	28.54	24.87	18.12	24.61

## Conclusion

In this paper, we applied the emerging deep learning technologies and collected traffic data occurred in urban to predict the traffic flow. Accurate traffic flow prediction can provide the useful information for urban operators to develop management measures or for residents to adjust their travel plans or routes. Furthermore, the study will enable cities to operate more efficiently and ultimately achieve the goal of an intelligent and sustainable city of the future. This research will contribute to the effective management of complex urban information. Specifically, in this paper, the Bi-GRU is applied to predict the traffic flow of urban expressways, and traffic flow data of four road sections are applied to the model for training and evaluation. Before model training, the ADF unit root test and differential processing are carried out for the four road sections for data stability. Moreover, ARIMA, LSTM, Bi-LSTM, and GRU, which has the ability to mine the temporal characteristics of traffic flow, are introduced for comparison to further evaluate the performance of the Bi-GRU model.

Through model training and validation, the overall prediction results of RMSE, MAPE and MAE of the Bi-GRU model are 30.38, 9.88% and 23.35, respectively. The prediction error of Bi-GRU is lower than that of other models, which indicates that the Bi-GRU model has the highest prediction performance. The traffic flow prediction errors of both Bi-LSTM and Bi-GRU are smaller than those of LSTM and GRU, which indicates that the bi-directional structure of mining historical and subsequent time series data is useful for traffic flow prediction. The prediction accuracy of deep learning methods (e.g. LSTM, Bi-LSTM, GRU, and Bi-GRU) is significantly higher than that of the traditional ARIMA model. The MAPE difference of Bi-GRU and GRU is 0.48% which is a small prediction error values. Therefore, the Bi-GRU is recommended for traffic flow prediction in scenarios with high prediction accuracy and limited road sections. GRU is recommended for predicting traffic flow in large road network scenarios, combining prediction accuracy and computational efficiency. In addition, this paper compares the prediction and observation of each road section. It concludes that the Bi-GRU model shows better prediction results during peak than low peak, and the proposed model has a certain lag.

There are some limitations of this study that need to be acknowledged. First, due to data limitations, we did not compare the prediction performance of Bi-GRU with the existing combination model (e.g., CNN-BiGRU (Meng, Toan, et al., 2022), DTW-BiGRU (Zou et al, 2020). Second, we should include more latest technologies in the comparison. In the future, we will apply the emerging technologies and construct the combined model to improve the model accuracy under different scenarios. Besides, we will show the prediction results for multiple methods.

## Data Availability

The datasets generated during and/or analysed during the current study are not publicly available due to privacy restrictions from the third party but are available from the corresponding author on reasonable request.
